# *N*-(4-Methyl-2-pyrid­yl)-*p*-toluidine

**DOI:** 10.1107/S160053680905586X

**Published:** 2010-01-16

**Authors:** Zainal Abidin Fairuz, Zaharah Aiyub, Zanariah Abdullah, Seik Weng Ng

**Affiliations:** a Department of Chemistry, University of Malaya, 50603 Kuala Lumpur, Malaysia

## Abstract

In the title compound, C_13_H_14_N_2_, the dihedral angle between the aromatic rings is 48.1 (1)° and the bridging C—N—C bond angle is 127.24 (12)°. In the crystal, intermolecular N—H⋯N hydrogen bonding about a center of inversion generates a hydrogen-bonded dimer.

## Related literature

For the structure of *N*-(2-pyrid­yl)-4-toluidine, see: Fairuz *et al.* (2008 ▶).
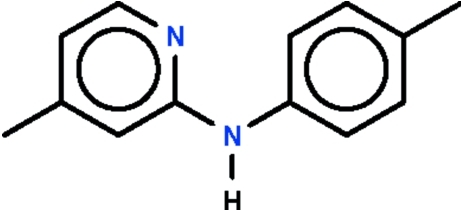


## Experimental

### 

#### Crystal data

C_13_H_14_N_2_
*M* *_r_* = 198.26
Monoclinic, 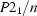

*a* = 10.9385 (11) Å
*b* = 7.5708 (8) Å
*c* = 13.4372 (14) Å
β = 95.246 (2)°
*V* = 1108.1 (2) Å^3^
*Z* = 4
Mo *K*α radiation
μ = 0.07 mm^−1^
*T* = 295 K
0.45 × 0.40 × 0.30 mm

#### Data collection

Bruker SMART APEX diffractometer
6758 measured reflections
2528 independent reflections
1797 reflections with *I* > 2σ(*I*)
*R*_int_ = 0.024

#### Refinement

*R*[*F* ^2^ > 2σ(*F* ^2^)] = 0.043
*wR*(*F* ^2^) = 0.142
*S* = 1.05
2528 reflections
143 parameters
1 restraint
H atoms treated by a mixture of independent and constrained refinement
Δρ_max_ = 0.17 e Å^−3^
Δρ_min_ = −0.13 e Å^−3^

### 

Data collection: *APEX2* (Bruker, 2008 ▶); cell refinement: *SAINT* (Bruker, 2008 ▶); data reduction: *SAINT*; program(s) used to solve structure: *SHELXS97* (Sheldrick, 2008 ▶); program(s) used to refine structure: *SHELXL97* (Sheldrick, 2008 ▶); molecular graphics: *X-SEED* (Barbour, 2001 ▶); software used to prepare material for publication: *publCIF* (Westrip, 2010 ▶).

## Supplementary Material

Crystal structure: contains datablocks global, I. DOI: 10.1107/S160053680905586X/xu2710sup1.cif

Structure factors: contains datablocks I. DOI: 10.1107/S160053680905586X/xu2710Isup2.hkl

Additional supplementary materials:  crystallographic information; 3D view; checkCIF report

## Figures and Tables

**Table 1:** Hydrogen-bond geometry (Å, °)

*D*—H⋯*A*	*D*—H	H⋯*A*	*D*⋯*A*	*D*—H⋯*A*
N1—H1⋯N2^i^	0.87 (1)	2.18 (1)	3.041 (2)	170 (2)

Symmetry code: (i) 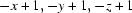.

**Fig. 1.**
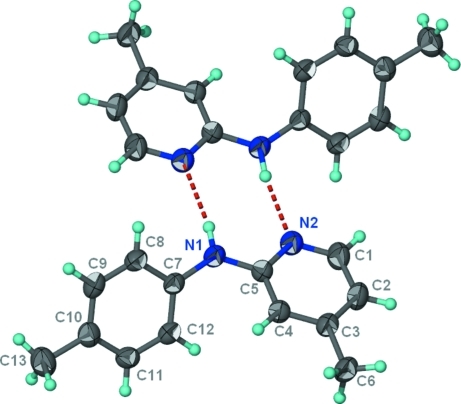
Thermal ellipsoid plot (Barbour, 2001) of the hydrogen-bonded C13H14N2 at the 50% probability level; hydrogen atoms are drawn as spheres of arbitrary radius. Dashed lines denote hydrogen bonds.

## supplementary crystallographic information

### Experimental

2-Chloro-4-methylpyridine (1 ml, 0.01 mol) and *p*-toluidine (1.2 g, 0.01 mol) were heated for 4 h. The product was dissolved in water and the solution extracted with ether. The ether extract was dried over sodium sulfate. Evaporation of the solvent gave large blocks of dark brown crystals. The crystals, when the outer parts were removed, were colorless.

### Refinement

Carbon-bound H-atoms were placed in calculated positions (C–H 0.93–0.96 Å) and were included in the refinement in the riding model approximation, with *U*(H) set to 1.2–1.5*U*(C). The amino H-atom was located in a difference Fourier map, and was refined with a distance restraint of N–H 0.86±0.01 Å; its temperature factor was refined.

### Crystal data

**Table:**

C_13_H_14_N_2_	*F*(000) = 424
*M**_r_* = 198.26	*D*_x_ = 1.188 Mg m^−^^3^
Monoclinic, *P*2_1_/*n*	Mo *K*α radiation, λ = 0.71073 Å
Hall symbol: -P 2yn	Cell parameters from 2289 reflections
*a* = 10.9385 (11) Å	θ = 2.5–28.1°
*b* = 7.5708 (8) Å	µ = 0.07 mm^−^^1^
*c* = 13.4372 (14) Å	*T* = 295 K
β = 95.246 (2)°	Irregular block, colorless
*V* = 1108.1 (2) Å^3^	0.45 × 0.40 × 0.30 mm
*Z* = 4	

### Data collection

**Table:**

Bruker SMART APEX diffractometer	1797 reflections with *I* > 2σ(*I*)
Radiation source: fine-focus sealed tube	*R*_int_ = 0.024
graphite	θ_max_ = 27.5°, θ_min_ = 2.3°
ω scans	*h* = −14→13
6758 measured reflections	*k* = −9→9
2528 independent reflections	*l* = −14→17

### Refinement

**Table:**

Refinement on *F*^2^	Secondary atom site location: difference Fourier map
Least-squares matrix: full	Hydrogen site location: inferred from neighbouring sites
*R*[*F*^2^ > 2σ(*F*^2^)] = 0.043	H atoms treated by a mixture of independent and constrained refinement
*wR*(*F*^2^) = 0.142	*w* = 1/[σ^2^(*F*_o_^2^) + (0.0692*P*)^2^ + 0.1529*P*] where *P* = (*F*_o_^2^ + 2*F*_c_^2^)/3
*S* = 1.05	(Δ/σ)_max_ = 0.001
2528 reflections	Δρ_max_ = 0.17 e Å^−^^3^
143 parameters	Δρ_min_ = −0.13 e Å^−^^3^
1 restraint	Extinction correction: *SHELXL97* (Sheldrick, 2008), Fc^*^=kFc[1+0.001xFc^2^λ^3^/sin(2θ)]^-1/4^
Primary atom site location: structure-invariant direct methods	Extinction coefficient: 0.044 (6)

### Fractional atomic coordinates and isotropic or equivalent isotropic displacement parameters (Å^2^)

**Table:**

	*x*	*y*	*z*	*U*_iso_*/*U*_eq_	
N1	0.51269 (11)	0.54344 (19)	0.64065 (9)	0.0571 (4)	
H1	0.4682 (13)	0.546 (2)	0.5832 (8)	0.063 (5)*	
N2	0.66732 (12)	0.46482 (18)	0.54571 (9)	0.0554 (4)	
C1	0.78715 (15)	0.4383 (2)	0.53779 (12)	0.0642 (5)	
H1A	0.8107	0.4072	0.4754	0.077*	
C2	0.87740 (14)	0.4535 (2)	0.61487 (13)	0.0619 (4)	
H2	0.9588	0.4288	0.6052	0.074*	
C3	0.84502 (13)	0.50645 (19)	0.70755 (11)	0.0490 (4)	
C4	0.72299 (13)	0.53940 (19)	0.71698 (11)	0.0471 (3)	
H4	0.6985	0.5788	0.7776	0.056*	
C5	0.63566 (13)	0.51384 (18)	0.63552 (10)	0.0458 (3)	
C6	0.93890 (14)	0.5264 (2)	0.79563 (12)	0.0607 (4)	
H6A	0.9168	0.6239	0.8360	0.091*	
H6B	1.0180	0.5479	0.7725	0.091*	
H6C	0.9418	0.4201	0.8347	0.091*	
C7	0.45006 (12)	0.55349 (19)	0.72722 (10)	0.0447 (3)	
C8	0.35038 (12)	0.6665 (2)	0.72803 (11)	0.0516 (4)	
H8	0.3294	0.7385	0.6731	0.062*	
C9	0.28208 (13)	0.6735 (2)	0.80914 (11)	0.0547 (4)	
H9	0.2153	0.7498	0.8077	0.066*	
C10	0.31070 (13)	0.5694 (2)	0.89293 (11)	0.0526 (4)	
C11	0.41089 (13)	0.4573 (2)	0.89151 (11)	0.0502 (4)	
H11	0.4322	0.3860	0.9467	0.060*	
C12	0.47974 (13)	0.44843 (19)	0.81091 (10)	0.0477 (4)	
H12	0.5464	0.3719	0.8124	0.057*	
C13	0.23520 (17)	0.5750 (3)	0.98106 (13)	0.0757 (5)	
H13A	0.1631	0.6452	0.9646	0.113*	
H13B	0.2829	0.6262	1.0373	0.113*	
H13C	0.2115	0.4572	0.9974	0.113*	

### Atomic displacement parameters (Å^2^)

**Table:**

	*U*^11^	*U*^22^	*U*^33^	*U*^12^	*U*^13^	*U*^23^
N1	0.0418 (7)	0.0866 (10)	0.0424 (7)	0.0084 (6)	0.0001 (5)	0.0006 (6)
N2	0.0492 (7)	0.0675 (8)	0.0496 (7)	0.0037 (6)	0.0048 (5)	−0.0037 (6)
C1	0.0536 (9)	0.0824 (12)	0.0583 (9)	0.0073 (8)	0.0136 (7)	−0.0093 (8)
C2	0.0426 (8)	0.0744 (11)	0.0698 (10)	0.0060 (7)	0.0104 (7)	−0.0040 (8)
C3	0.0433 (8)	0.0435 (8)	0.0599 (9)	−0.0004 (6)	0.0025 (6)	0.0045 (6)
C4	0.0439 (7)	0.0494 (8)	0.0479 (7)	0.0008 (6)	0.0049 (6)	−0.0010 (6)
C5	0.0427 (7)	0.0476 (8)	0.0471 (7)	0.0022 (6)	0.0049 (6)	0.0029 (6)
C6	0.0435 (8)	0.0664 (10)	0.0706 (10)	−0.0005 (7)	−0.0032 (7)	0.0044 (8)
C7	0.0363 (7)	0.0533 (8)	0.0434 (7)	−0.0015 (6)	−0.0023 (5)	−0.0024 (6)
C8	0.0424 (7)	0.0570 (9)	0.0542 (8)	0.0033 (6)	−0.0022 (6)	0.0070 (7)
C9	0.0411 (7)	0.0585 (9)	0.0643 (9)	0.0052 (6)	0.0031 (6)	−0.0040 (7)
C10	0.0452 (8)	0.0625 (9)	0.0500 (8)	−0.0080 (7)	0.0034 (6)	−0.0102 (7)
C11	0.0479 (8)	0.0559 (9)	0.0450 (7)	−0.0082 (6)	−0.0047 (6)	0.0031 (6)
C12	0.0408 (7)	0.0497 (8)	0.0512 (8)	0.0021 (6)	−0.0036 (6)	−0.0022 (6)
C13	0.0698 (11)	0.0969 (14)	0.0623 (10)	−0.0047 (10)	0.0172 (8)	−0.0109 (10)

### Geometric parameters (Å, °)

**Table:**

N1—C5	1.3716 (18)	C6—H6C	0.9600
N1—C7	1.4051 (18)	C7—C8	1.3867 (19)
N1—H1	0.875 (9)	C7—C12	1.391 (2)
N2—C1	1.340 (2)	C8—C9	1.378 (2)
N2—C5	1.3380 (18)	C8—H8	0.9300
C1—C2	1.369 (2)	C9—C10	1.386 (2)
C1—H1A	0.9300	C9—H9	0.9300
C2—C3	1.385 (2)	C10—C11	1.388 (2)
C2—H2	0.9300	C10—C13	1.505 (2)
C3—C4	1.375 (2)	C11—C12	1.376 (2)
C3—C6	1.502 (2)	C11—H11	0.9300
C4—C5	1.399 (2)	C12—H12	0.9300
C4—H4	0.9300	C13—H13A	0.9600
C6—H6A	0.9600	C13—H13B	0.9600
C6—H6B	0.9600	C13—H13C	0.9600
			
C5—N1—C7	127.24 (12)	C8—C7—C12	118.16 (13)
C5—N1—H1	115.4 (11)	C8—C7—N1	118.89 (13)
C7—N1—H1	117.1 (11)	C12—C7—N1	122.87 (13)
C1—N2—C5	116.68 (13)	C9—C8—C7	120.90 (13)
N2—C1—C2	124.77 (15)	C9—C8—H8	119.5
N2—C1—H1A	117.6	C7—C8—H8	119.5
C2—C1—H1A	117.6	C8—C9—C10	121.47 (14)
C1—C2—C3	118.61 (14)	C8—C9—H9	119.3
C1—C2—H2	120.7	C10—C9—H9	119.3
C3—C2—H2	120.7	C9—C10—C11	117.16 (14)
C4—C3—C2	117.75 (14)	C9—C10—C13	121.57 (15)
C4—C3—C6	120.58 (14)	C11—C10—C13	121.26 (15)
C2—C3—C6	121.67 (14)	C12—C11—C10	122.03 (14)
C3—C4—C5	120.13 (13)	C12—C11—H11	119.0
C3—C4—H4	119.9	C10—C11—H11	119.0
C5—C4—H4	119.9	C11—C12—C7	120.27 (13)
N2—C5—N1	115.25 (12)	C11—C12—H12	119.9
N2—C5—C4	121.97 (13)	C7—C12—H12	119.9
N1—C5—C4	122.73 (13)	C10—C13—H13A	109.5
C3—C6—H6A	109.5	C10—C13—H13B	109.5
C3—C6—H6B	109.5	H13A—C13—H13B	109.5
H6A—C6—H6B	109.5	C10—C13—H13C	109.5
C3—C6—H6C	109.5	H13A—C13—H13C	109.5
H6A—C6—H6C	109.5	H13B—C13—H13C	109.5
H6B—C6—H6C	109.5		
			
C5—N2—C1—C2	1.7 (3)	C5—N1—C7—C8	146.94 (15)
N2—C1—C2—C3	−2.5 (3)	C5—N1—C7—C12	−36.3 (2)
C1—C2—C3—C4	0.6 (2)	C12—C7—C8—C9	−0.5 (2)
C1—C2—C3—C6	−179.91 (16)	N1—C7—C8—C9	176.39 (13)
C2—C3—C4—C5	2.0 (2)	C7—C8—C9—C10	0.5 (2)
C6—C3—C4—C5	−177.51 (13)	C8—C9—C10—C11	−0.2 (2)
C1—N2—C5—N1	178.72 (14)	C8—C9—C10—C13	−179.16 (15)
C1—N2—C5—C4	1.1 (2)	C9—C10—C11—C12	0.0 (2)
C7—N1—C5—N2	163.28 (14)	C13—C10—C11—C12	178.95 (14)
C7—N1—C5—C4	−19.1 (2)	C10—C11—C12—C7	0.0 (2)
C3—C4—C5—N2	−3.0 (2)	C8—C7—C12—C11	0.3 (2)
C3—C4—C5—N1	179.59 (14)	N1—C7—C12—C11	−176.47 (13)

### Hydrogen-bond geometry (Å, °)

**Table:**

*D*—H···*A*	*D*—H	H···*A*	*D*···*A*	*D*—H···*A*
N1—H1···N2^i^	0.87 (1)	2.18 (1)	3.041 (2)	170 (2)

Symmetry codes: (i) −*x*+1, −*y*+1, −*z*+1.
